# Grain Refinement Caused by Larger Particles in Laser 3D Printing of AISI 304L Stainless Steel

**DOI:** 10.3390/ma18061223

**Published:** 2025-03-10

**Authors:** Xuhuai Zhang, Xing Lu

**Affiliations:** School of Material Science and Engineering, Dalian Jiaotong University, Xi’an Road Street, Shahekou District, Dalian 116028, China

**Keywords:** laser 3D printing, stainless steel, particle size, grain refinement, mechanical property

## Abstract

Additively manufactured (AM) stainless steel has attracted a lot of attention for its competitive performance advantages over parts prepared by traditional methods. However, the influence of the powder characteristic of AISI 304L stainless steel on the laser 3D printing (3DP) process has yet to be clarified. In this research, the effect of the particle size of atomized AISI 304L stainless steel powder on 3DP of a powder-fed laser was studied, the grain morphology of different printed samples was analyzed by electron backscatter diffraction (EBSD) technology, and the mechanical properties were investigated via quasi-static tensile experiments. It was found that the use of small particles made the columnar crystal area mix with fine equiaxed grains in single-pass laser melting deposition, resulting in an obvious grain refinement effect. However, in multi-pass deposition, samples deposited with small particles exhibited more significant grain coarsening and anisotropy effects than those of larger particles, resulting in a significant reduction in plasticity. This can be attributed to the grain merging and growth mechanism caused by the thermal shock effect of multi-pass laser deposition, which is controlled by the grain configuration formed in the early-pass deposition. The results show that the use of powder particles greater than 50 μm is of great significance for improving the quality of AISI 304L stainless steel 3DP products.

## 1. Introduction

Additive manufacturing (AM), also known as three dimensional printing (3DP), is widely recognized as a revolutionary component processing technology that can enhance the competitiveness of the manufacturing industry [1]. In terms of metal 3D printing, powder bed fusion and directed energy deposition are the two most commonly used AM technologies in industrial applications, both of which utilize metal powders as raw materials. It is widely accepted that the quality of powder used in AM is a key factor affecting process performance and final component quality [2]. Therefore, only by reducing the cost of producing metal powders and fully understanding the impact of powders on process performance can the widespread application of additive manufacturing technology in modern industry be promoted more quickly.

The effect of raw powder on AM includes two different categories, the chemical aspect of the powder surface and the physical properties of the powder itself. The chemical aspect refers to the formation of oxidizing surfaces or other impurities on the surface of the powder, which are introduced into the AM part to reduce its quality [3]. Physical properties refer to the characteristics of flowability, particle size distribution (PSD), internal porosity, etc., which are critical to the quality of the part. Since different AM equipment uses different powder supply and dispensing methods, the optimal powder characteristics are also different.

The PSD of used powder significantly affects the grain size and the microstructure formed during laser 3DP. For particles with a diameter of less than 30 μm, the particles tend to form agglomerates due to the gravitational action dominated by the adhesion force, resulting in an increase in the surface roughness of the deposited layer [4]. At the same time, small particle size powders provide a larger surface area to facilitate heat conduction but, due to their poor flowability, they are prone to irregular build-up during laser powder bed fusion (LPBF), which in turn affects the quality of the final part [5]. For large-size powders, the remaining large-size powders can significantly reduce the surface roughness of the deposited layer when particles with a diameter of less than 60 μm are removed [4]. In addition, large particle size powders can improve the uniformity of powder spreading to a certain extent due to their better fluidity and lower agglomeration tendency, thereby reducing porosity and optimizing grain structure [6].

AISI 304L stainless steel (304L SS), also known as ultra-low carbon stainless steel, has been widely used in nuclear reactor components, chemical processing equipment, and the petroleum industry due to its excellent corrosion resistance, high oxidation resistance, and excellent mechanical properties [7]. In addition, 304L SS is a promising candidate for oxide dispersion-reinforced steel matrices, which are expected to achieve excellent comprehensive properties such as good mechanical properties, high corrosion resistance, and resistance to high-temperature oxidation [8]. Recently, the AM of 304L SS parts has also attracted a lot of attention, and most of the current literature reports focus on the effect of processing parameters on mechanical properties and corrosion resistance [9,10,11,12,13]. For example, Mahmood et al. found that laser scanning speed and laser power were inversely proportional to the average grain size, while there was a direct relationship between the powder feed rate and the average grain size [14]. However, there are few studies on the relationship among powder properties, mechanical properties, and the microstructure of 304L SS stainless steel. Therefore, it is important to establish a link between the properties of 304L SS powder and the performance of the AM final part.

In this research, we systematically studied the effects of the PSD of commercial 304L SS stainless steel powders on the microstructure and mechanical properties of the 3DP samples. The grain refinement behavior of single-pass laser deposition and grain roughening behavior in multi-pass laser deposition were discussed.

## 2. Materials and Methods

Commercial 304L SS stainless steel powder prepared by nitrogen atomization (Zhuohang Metal, Xingtai, China) was used as the 3DP raw material. In order to investigate the effect of powder particle size on the microstructure and properties of the 3DP samples, the as-received powder was carefully sieved using stainless steel sieves with a mesh size of 150–320. Powders with mesh sizes of 150–170, 170–230, 230–270, and 270–320 were collected and named as Sample A, Sample B, Sample C, and Sample D, respectively. A laser particle size analyzer (Haoyu, HYL-2080, Dandong, China) was used to test the PSD of the sieved powder samples, using deionized water as a dispersant, with a testing time of 30 s.

**Figure 1:**
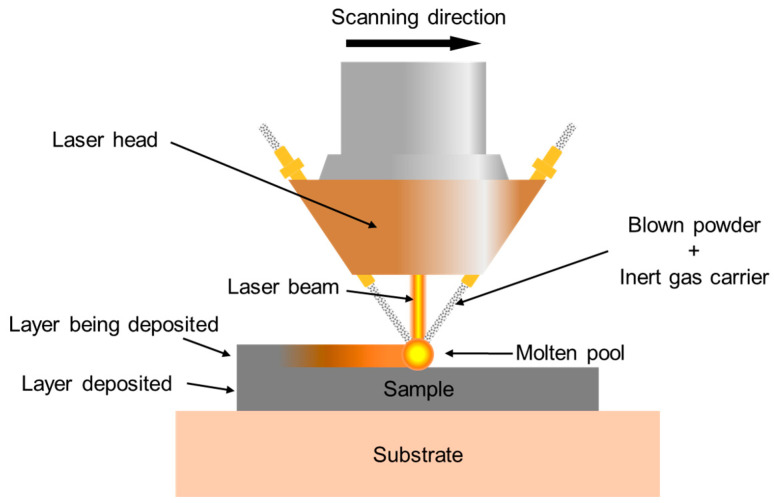
Schematic diagram of the powder feeding laser 3D printing device.

A powder-fed laser melting deposition (also 3DP) device was used to prepare different samples. The schematic diagram of the equipment is shown in Figure 1, and the printing process parameters are shown in Table 1. The cut 304L SS steel plates with a size of about 50 mm × 50 mm × 10 mm were used as the 3DP substrates. For single-pass laser deposition, a sample with a length of about 20 mm was deposited on the substrate. For multi-pass laser deposition samples, a 42-pass process was used, stacked with 6 layers up to 13 mm in height and 45 mm in length.

A wire cutting machine was used to cut the printed sample into different sizes for structural and property analyses. A metallurgical microscope (Zeiss, Axio Observer, Munich, Germany) was used to observe the microstructure of the cross-section of the sample. Tensile tests of the printed samples were performed on a servohydraulic testing machine (Instron 5982, Boston, MA, USA) at a strain rate of 5 × 10^−4^ s^−1^. Thin plate samples with typical dog-bone shape, with effective test area dimensions of 1 mm × 1 mm × 7 mm, were used for tensile experiments, following the ASTM E8 standard. At least 5 repeated tensile experiments were performed on each sample to obtain reliable data. The fracture surface was examined using a field emission gun scanning electron microscope (Fe-SEM, Zeiss Gemini SEM 360, Oberkochen, Germany). In order to analyze the grain morphology, electron backscatter diffraction (EBSD) images of different samples were taken with an SEM (Zeiss Jena, Supra 55 SEM, Jena, Germany). The foil samples for EBSD analysis were cut from the cross-section of the deposited samples, and subsequently mechanically polished, then followed by electro-polishing under a voltage of 15 V for ~20 s at room temperature. The working distance used for the EBSD measurement was about 13 mm, the scanning step size was 5 μm, the accelerating voltage was 20.00 kV, and the tilt angle of the sample was 70.00°.

## 3. Results

**Figure 2:**
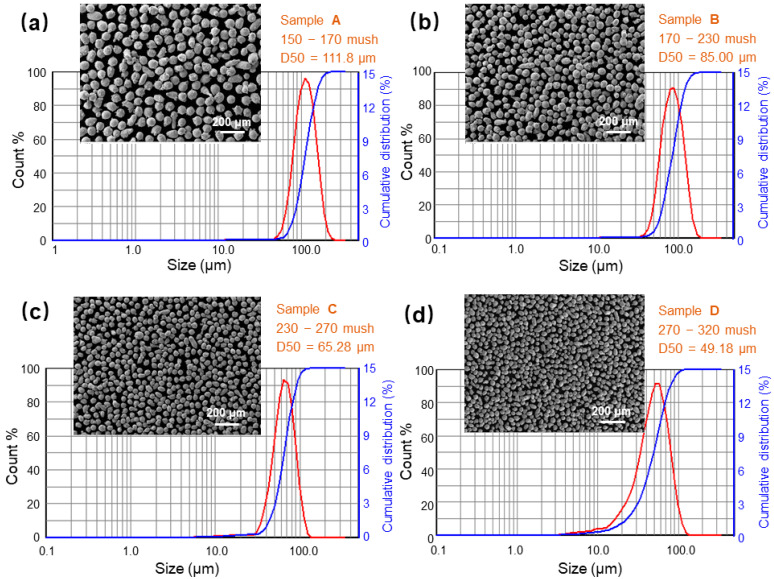
SEM images and particle size distribution curves of different particle samples: (**a**–**d**) samples A, B, C, and D, respectively.

Figure 2 displays the SEM images and the analysis results of various powder samples using a laser particle size analyzer. After passing through some special stainless steel mesh screens of 150, 170, 230, 270, and 320 mesh sizes, the particle size of 304L SS stainless steel powder becomes remarkably uniform. The average diameter (D50) values of samples A, B, C, and D, measured using the laser particle size analyzer, were 118.8, 85, 65.28, and 49.18 μm, respectively. Meanwhile, the specific surface areas were 21.09, 27.81, 37.82, and 58.75 m^2^/kg, respectively. Notably, the specific surface area of Sample D is 278% of that of Sample A, suggesting a potentially higher level of oxide impurities adsorbed on the powder surface.

**Figure 3:**
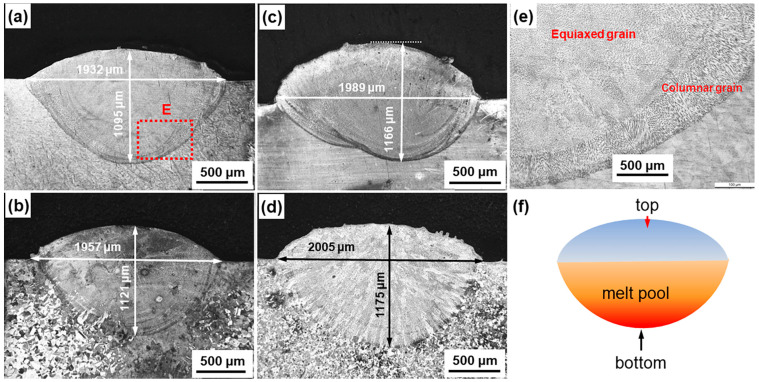
Metallographic photographs of cross-sections of single-pass laser-deposited samples using raw materials with different particle sizes of: (**a**–**d**) powder A–D; (**e**) enlarged view of the dashed box E area in Figure 3a; (**f**) schematic diagram showing the shape of the molten pool.

The microstructure of laser 3DP materials is primarily formed through the solidification of the laser melt pool. Therefore, the size, shape, and cooling conditions of the laser melt pool are important factors that must be considered. Figure 3a–d show the metallographic images of the cross-section of the deposited samples by the single-pass laser deposition using raw powders with different sizes. It shows that the shape of the molten pool formed by laser heating is close to oval, and its width and depth exhibit obvious dependence on the particle size of the powder. As the particle size decreases, the width of the melt pool increases from about 1932 μm to 2005 μm, and the depth also increases from about 1095 μm to 1175 μm. It is suggested that the smaller the powder size, the larger the melt pool formed. In our opinion, it is related to the change in the number of particles and surface area caused by the change in the particle size of the raw powder. According to the geometric characteristics of spherical particles, the relationship between the particle size *d* and the number of particles *N* under the same total volume *V_total_* is as follows:

(1) $$N=\frac{6V_{total}}{\pi d^{3}}$$

Assuming that the powder volume is 1 m^3^, the number of particles are 1367, 3111, 6869, and 16064 when the particle sizes are 111.8 μm, 85 μm, 65.28 μm, and 49.18 μm, respectively. It can be seen that the reduction in particle size by half and the increase in the number of particles by more than 10 times will significantly increase the total surface area of particles. In this study, Sample D has more particles and greater specific surface area under the condition of consistent powder delivery, which will melt at a faster rate in laser heating to undergo superheating, resulting in a larger melt pool.

Figure 3f shows a schematic of the laser melt pool in this study. The cooling conditions at the bottom and top of the melt pool are different; the bottom cools quickly and has directionality, while the top cools quickly but lacks directionality. As a result, columnar grains typically form at the bottom of the melt pool, while equiaxed grains mainly form at the top and the center, as shown in Figure 3e.

**Figure 4:**
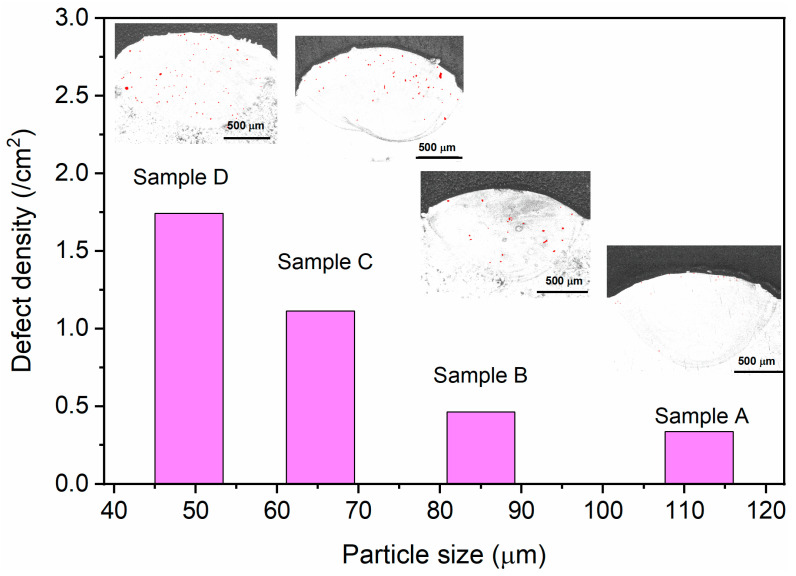
Powder particle size vs. defect density in single-pass laser 3D printing samples, with the insets showing the defect distribution.

The metallographic photographs shown in Figure 3a–d also show the presence of casting defects. After staining, the distribution of defects such as cast pores in different samples can be more clearly observed, as shown in the insets in Figure 4. Obviously, samples prepared from small-sized powders contain a higher number of defects, with defect densities of up to 1.74 cm^−2^ in the cross-section (Sample D). With the increase in particle size, the defect density decreases significantly, and when the particle size is 111.8 μm (Sample A), the defect density is only 0.34 cm^−2^, which is 80% smaller than that of Sample D. It shows that the use of larger particles can effectively reduce the defects of 3DP products.

**Figure 5:**
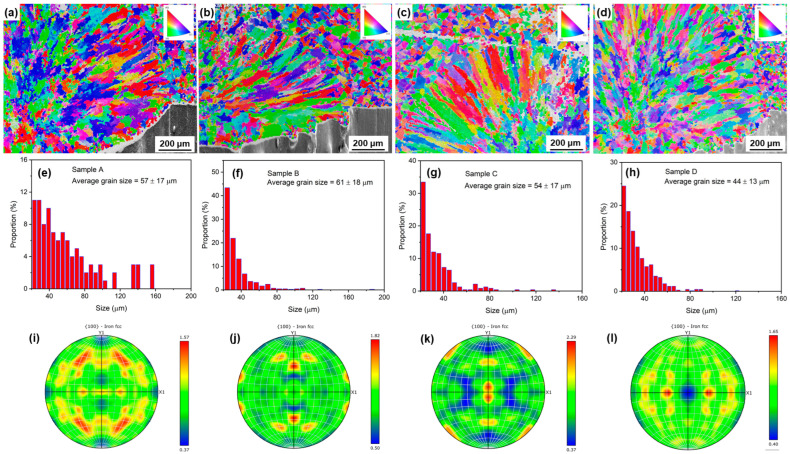
EBSD analyses of samples deposited by single-pass laser deposition using powders A, B, C, and D, respectively: (**a**–**d**) EBSD images; (**e**–**h**) grain size distributions; (**i**–**l**) $\langle 100\rangle$ pole diagrams of FCC Fe.

To more clearly analyze the effect of particle size on the solidification behavior of the laser melt pool, typical electron backscatter diffraction (EBSD) technology was used to analyze the grain morphology of the samples prepared using different powders after a single pass of printing, as shown in Figure 5a–d.

In general, with the decrease in the average particle size of the raw powder, the proportion of equiaxed grains in a single melt channel gradually increases, and the epitaxial growth of columnar grains to the equiaxed grain region in the melt channel of the finest powder is obvious. Figure 5d,h show the grain features of a sample prepared with a size of ~44 μm, where the diameter of the columnar grains is significantly smaller than that of the samples prepared from larger particle size raw powder (Figure 5e–g). However, there is no significant change in the length of the columnar grains in the samples printed with different sizes of raw powder. In addition, there is a transition zone between the equiaxed grains at the top of the melt pool and the columnar grains at the bottom, which is mixed with columnar grains and equiaxed grains. Interestingly, when the powder particle size is minimized, this transition zone significantly increases, and even a certain amount of equiaxed crystals are mixed throughout the entire columnar grain region. This may be related to the special flowability and the melting behavior of small-sized particles.

Figure 5i–l show the pole plots of different samples by single-pass laser deposition. It shows that there is no obvious preferred orientation in the <100> direction for all the samples, which is related to the underdeveloped columnar grains.

**Figure 6:**
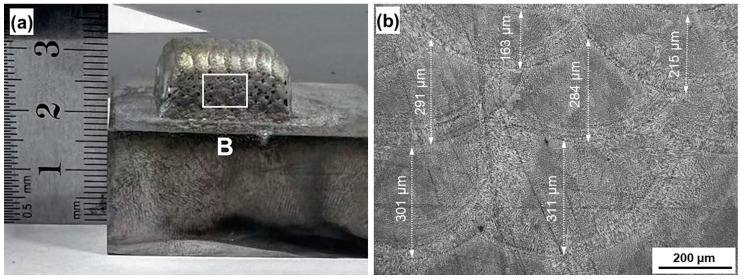
(**a**) Typical macroscopic optical photograph of the bulk samples deposited through multi-pass laser deposition using A powder; (**b**) metallographic photograph of the sample taken from position B indicated by the white square in Figure 6a.

In laser 3DP, the component parts are stacked layer by layer, and the part that is formed first is generally subjected to repeated thermal shocks caused by the melt pool of the later laser heating, causing some changes in the microstructure. Figure 6a shows an optical photograph of the sample prepared for this study, which is based on a thick stainless steel plate with good heat dissipation. In a cross-section perpendicular to the substrate (as shown in the white box B in Figure 6a), there is typically a stacked structure formed by a multi-pass laser melt pool, with columnar and equiaxed grains crossed together, as shown in Figure 6b. The depth of the melt pool produced by the multi-pass laser heating is theoretically the same as that of the single-pass laser heating due to the same amount of powder fed and laser power. However, we observed that the depth decreases after repeated melting and heating in multi-pass laser deposition, ranging from 163 μm to 311 μm, which is about ~1/3 of that of the single-pass melt pool, as shown in Figure 5b. It means that the top of the solidified melt pool is melted 2/3 by the later multi-pass laser heating, i.e., the equiaxed crystal area is essentially re-melted. At the same time, the un-melted and residual part of the solidified melt pool is also subjected to the high-temperature heating and annealing process close to the melting point, which will inevitably have a great impact on the microstructure and mechanical properties.

**Figure 7:**
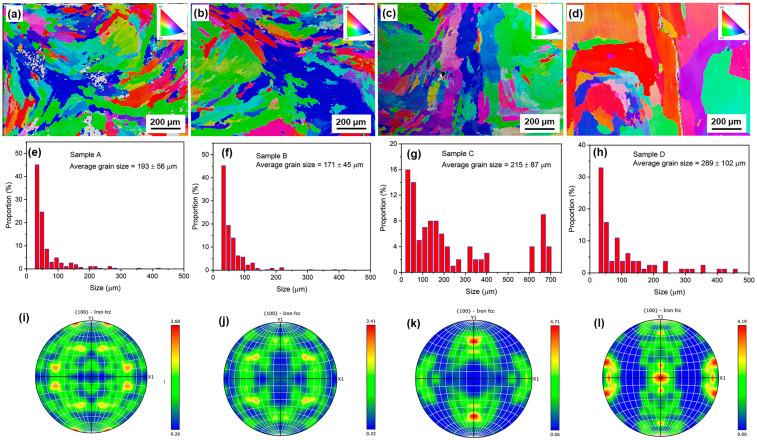
EBSD analyses of the bottoms of bulk samples deposited through multiple passes using powders of different particle sizes, namely samples A, B, C, and D: (**a**–**d**) EBSD images; (**e**–**h**) grain size distributions; (**i**–**l**) $\langle 100\rangle$ pole diagrams of FCC Fe.

The bottom layer, close to the substrate, was subjected to the highest number of thermal shocks. Therefore, we took EBSD images of the samples at this location for the four samples, as shown in Figure 7a–d. Compared to the single-pass laser deposition sample shown in Figure 5a–d, the columnar grain diameter at the bottom of the multi-pass deposition sample is significantly larger. This is due to the fact that in the multi-pass deposition process, the part that solidifies first is repeatedly heated, causing repeated grain growth.

Figure 7a–d also show the effect of the particle size of the powder feedstock on the micromorphology of the columnar crystal region at the bottom of the multi-pass deposited sample. Of particular note, it can be found that the diameter of the columnar grains in the multi-pass sedimentation samples increases significantly as the particle size decreases (Figure 7e–h). For laser-deposited samples with a particle size of less than 50 μm, the diameter of the columnar crystals grows to more than a few hundred microns. It not only shows that the influence of laser thermal shock on the microstructure of 3DP stainless steel is too large to be ignored, but also proves that the effect of raw material particle size on the grain size of multi-pass laser deposition samples is exactly the opposite of that of single-pass deposition.

Figure 7i–l show the pole plots of different samples. For samples deposited with large-sized particles, there is no obvious preferred orientation in the <100> direction (Figure 7i–j). However, for samples C and D with smaller particle sizes, the preferred orientation in the <100> direction is significantly strengthened, which can be attributed to the appearance of coarse columnar grains.

**Figure 8:**
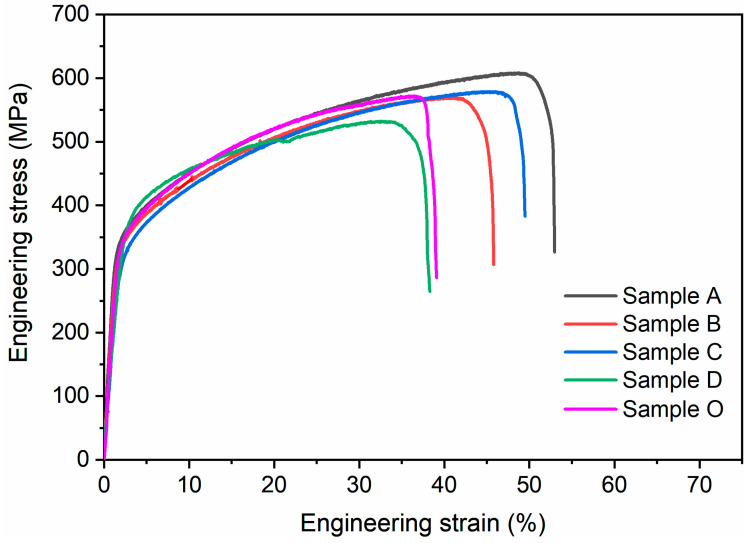
Tensile stress–strain curves of the samples prepared using powders of different particle sizes.

Figure 8 shows the tensile stress–strain curves of bulk samples prepared from powders with different particle sizes, and the corresponding mechanical properties are shown in Table 2. All the samples in the present study show good plasticity and better tensile strength than the samples obtained by conventional manufacturing methods. For example, the yield strength (σ_0.2_, at 0.2% offset) of Sample A prepared with large particles is measured to be 335 ± 5 MPa, which is about 1.6 times higher than that of the dislocation-free 304L SS stainless steel sample (210 MPa) [15]. It is well known that the concept of stacking fault energy is important in austenitic steels, which influences the plastic deformation mechanisms such as transformation-induced plasticity, twinning-induced plasticity, and microband-induced plasticity [16,17,18]. Laser 3DP has ultra-fast heating and cooling rates, which can lead to a higher presence of dislocations and residual stresses in the solidified microstructure, thereby increasing the stacking fault energy and contributing to the enhancement of strength. However, with the decreased particle size, the plasticity is reduced. We believe this phenomenon relates to the complex microstructure of the 3DP sample after repeated thermal shocks. The use of small-particle raw materials generally brings more physicochemical and mechanical defects and more uneven grains to the printed product, which brings certain interference to the plastic deformation, resulting in the plasticity of the sample being significantly smaller than that of the large-particle sample.

In addition, the 3DP samples with the widest range of particle size distribution before sieving were also subjected to tensile testing for comparison. Interestingly, its strength is similar to that of the large particle sample (samples A and B), while its plasticity is similar to that of the small particle sample (sample D).

**Figure 9:**
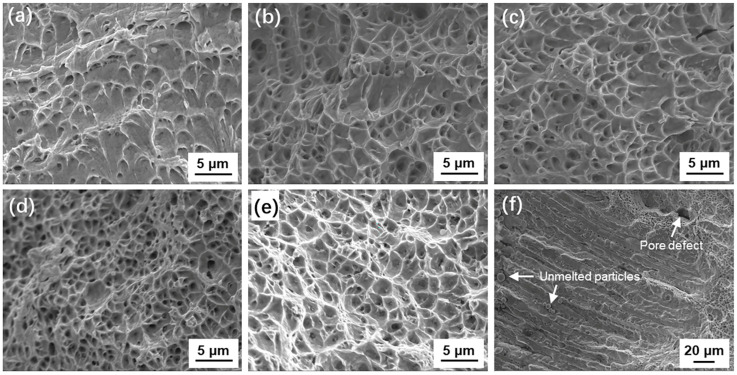
(**a**–**e**) SEM images of the fracture surfaces of Sample A–O; (**f**) typical SEM image of the fracture surface of columnar crystal region in Sample D.

304L SS stainless steel is a typical ductile metal material that usually has good plasticity, and its fracture exhibits toughness characteristics. Figure 9a–d compare the fracture morphology of Sample A–D, both of which have typical dimple characteristics, suggesting that they share ductile fracture behavior. For dimple fractures, a larger dimple generally represents better plasticity in ductile metals [19]. This is because the dimples are essentially deformation holes formed on the fracture surface of ductile materials. When subjected to greater plastic deformation, the holes grow bigger to form a larger size dimple. Herein, Figure 9a–c show typical larger dimple features, which are consistent with the better plasticities of Sample A–C. For Sample D (Figure 9d), the dimple size is significantly smaller than those of Sample A–C, and even smaller than that of Sample O with the full particle size range (Figure 9e, un-sieved powder), indicating that the 3DP product with small particles has a relatively brittle deformation behavior. It is important to note that the coarse columnar crystals contained in Sample D are often the source of cracks, as shown in Figure 9f. This is due to the fact that the heat conduction of the fine raw powder is not smooth and the heating is uneven, and some of the un-melted raw material particles are accumulated at the interface front and frozen in the 3DP part. These un-melted particles, along with another casting defect that tends to segregate at the interface, are prone to crack initiation and are responsible for the deterioration in plasticity.

Table 2 also lists the Vickers hardness test results of different samples. The Vickers hardness values of single-pass deposition samples using different particle sizes of raw materials are 223–230 MPa, with small differences, which is consistent with the grain size results. After multiple deposition processes, the hardness value of the sample significantly decreased compared to that of single-pass deposition processes, and the hardness of the deposited sample gradually decreased as the particle size of the raw material decreased. This is because the smaller the raw material particles, the smaller the grain size deposited in a single pass, resulting in more obvious grain growth during the multi-pass laser melting process, thereby causing a decrease in hardness. In addition, it is known that there is a strong correlation between hardness and the fatigue strength of materials [20]:

(2) $$\sigma_{w}=1.6\;HV\pm 0.1\;HV$$

where $\sigma_{w}$ is the fatigue limit, and HV is Vickers hardness. By substituting the hardness data of the multi-pass samples in Table 2 into Equation (2), the fatigue limits of samples A, B, C, D, and O can be evaluated to be 334.1, 302.2, 294.9, 272.2, and 306.1 MPa, respectively. It is demonstrated that using larger particles is beneficial for obtaining 3DP products with high fatigue performance.

## 4. Discussion

The influence of the particle size of raw material powder on the quality of parts and components is one of the key process parameters of laser 3D printing technology. In addition, scan speed and laser power are also key parameters to control grain size and porosity. For example, with a fixed laser power, decreasing the scanning speed allows more particles to bind and form a more contiguous region, thus reducing porosity [21]. However, if the laser power is reduced or the scanning speed is increased, there may be incomplete or partially melted particles in the melt pool that act as new nuclei to promote grain refinement [22].

Under the condition of fixed scanning speed and laser power, it was found that the size of the powder particles also showed a significant adjustment effect on the morphology and size of the solidified grains. The columnar crystal region is mixed with a large number of small equiaxed grains, which can be attributed to the following aspects. Firstly, small particles generally show a higher tendency to agglomerate, which not only reduces the fluidity of the powder but also causes the particles inside the agglomerated clusters to melt incompletely, thus acting as a natural crystal nucleus and refining the grains. Secondly, the specific surface area of small particles is large and the laser absorption rate is high, which may lead to local over melting or porosity. Thirdly, the agglomeration of small particles causes uniformity of particle size distribution, which affects the consistency of the melt pool and increases spatter and defects. Fourthly, small particles have large differences in heat conductivity, and the instability of the melt pool induces faster cooling rates and greater supercooling, which refines the grains and may lead to higher residual stresses.

Another issue that needs to be clarified is the mechanism of grain growth, especially columnar grains, in multi-pass laser deposition. The driving force for grain growth in the first solidified part under the thermal shock of the later laser pass may come from the residual stress formed by the rapid cooling of the laser forming itself and the high interfacial energy provided by the initial microstructure. For residual stresses, Sample D using small particle feedstock has a more complex grain morphology, which usually means that it has greater residual stress. As a result, Sample D has a greater tendency to grow during multi-pass laser deposition. For the interfacial energy, we believe that the interfacial energy provided by different grain configurations in this research plays a major role in the growth of columnar grains.

**Figure 10:**
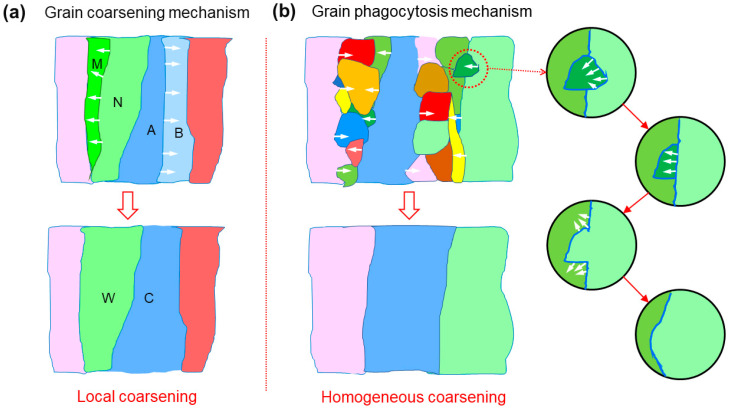
(**a**) Schematic diagram describing the grain coarsening mechanism, in which two grains with similar orientations colored in different shades of blue (A, B) and green (M, N) preferentially merge and grow (C and W); (**b**) schematic diagram describing the grain phagocytosis mechanism derived from the interface behavior between grains with significant size differences.

When the lattice orientation difference between adjacent columnar grains is small, their grain boundaries easily form a co-lattice or near-co-lattice relationship, so they can grow through the grain coarsening mechanism. As shown in Figure 10a, if the difference in atomic orientation between grains A and B is very small, the A/B interface must consist of sparse dislocations. When the temperature rises to a certain range due to thermal shock, the dislocations on the A/B interface can migrate elsewhere through slip and climbing, causing the grain boundaries between grain A and B to disappear and merge into one big columnar grain C. Obviously, this growth mode depends on the crystal orientation of adjacent grains, and the randomness is large, so it results in uneven local growth.

In the case of Figure 5d, the presence of a large number of grain boundaries results in high interfacial energy of the whole system. In particular, some small equiaxed grains are embedded inside the columnar crystals, as shown in Figure 10b. When the temperature rises to a high level due to thermal shock, the curved grain boundaries convex into the columnar grain are flattened under the action of interfacial tension, resulting in the growth of the columnar grain. Its growth form is carried out in the way that large grains swallow small grains, so it is called the grain phagocytosis mechanism, which corresponds to the uniform growth/coarsening of columnar grains.

In this study, an interesting phenomenon was found, that is, the use of fine particles could refine the microstructure of single-pass laser deposition but caused the coarsening of multi-pass laser deposition microstructure. It fully demonstrates the complexity of the influence of powder particle size distribution on the laser melting deposition process. Therefore, the findings of this study are of great significance for improving the performance of laser 3D printing or laser cladding coating products.

## 5. Conclusions

In the present study, the effects of powder particle size on the microstructure and mechanical properties of laser 3DP 304L SS stainless steel were studied for powder feeding laser melting deposition technology. The results show that the powder particle size has a significant effect on the grain micromorphology of laser 3DP 304L SS stainless steel. With decreases in powder particle size, the microstructure of single-pass laser 3DP tends to be refined, but the coarsening of columnar crystals is induced in the microstructure of multi-pass laser 3DP. This is related to the repeated thermal shocks caused by multi-pass laser melting. The cyclic temperature rises, and complex grain boundary configurations play important roles in grain engulfment and growth. The results of this study clarify the effect of powder particle size on the grain refinement effect of laser 3DP 304L SS stainless steel, deepening the understanding of the solidification process of laser 3DP metal.

## Figures and Tables

**Table 1:** The process parameters of powder-fed laser 3D printing selected for this study.

Processing Parameter	Value
Laser power (P)	1100 W
Laser scan speed (v)	480 mm/min
Layer height	1.5 mm
Power feed rate	9.3 g/min
Dwell time between each fill run	30 s
Laser beam diameter (D)	3 mm
Linear energy density (E = P/vD)	91.6 J/mm^2^

**Table 2:** Mechanical properties of different samples using raw particles with varying particle sizes.

Sample Name	Particle Size (μm)	Yield Strength (MPa)	Tensile Strength (MPa)	Tensile Plasticity (%)	Single-Pass Hv (MPa)	Multi-Pass Hv (MPa)
A	118.8	335 ± 5	610 ± 15	51.2 ± 1.6	228 ± 2	209 ± 7
B	85	315 ± 9	570 ± 12	44.2 ± 2.2	230 ± 4	189 ± 3
C	65.28	307 ± 12	580 ± 10	47.2 ± 3.5	226 ± 5	184 ± 4
D	49.18	365 ± 13	532 ± 18	36.8 ± 4.8	223 ± 4	170 ± 3
O	82.7	322 ± 7	572 ± 16	37.9 ± 3.9	224 ± 8	191 ± 4

## Data Availability

Data presented are original and not inappropriately selected, manipulated, enhanced, or fabricated. Any reader interested in this study can contact the author to obtain the original data.
